# Metformin-induced suppression of *IFN-α via* mTORC1 signalling following seasonal vaccination is associated with impaired antibody responses in type 2 diabetes

**DOI:** 10.1038/s41598-020-60213-0

**Published:** 2020-02-24

**Authors:** Wipawee Saenwongsa, Arnone Nithichanon, Malinee Chittaganpitch, Kampaew Buayai, Chidchamai Kewcharoenwong, Boonyarat Thumrongwilainet, Patcharavadee Butta, Tanapat Palaga, Yoshimasa Takahashi, Manabu Ato, Ganjana Lertmemongkolchai

**Affiliations:** 1 Centre for Research and Development of Medical Diagnostic Laboratories, Faculty of Associated Medical Sciences, Khon Kaen University, Khon Kaen, Thailand; 2 Disease Prevention and Control Region 10th, Ubonratchathani, Ministry of Public Health, Mueang Nonthaburi, Thailand; 3 National Influenza Centre, Department of Medical Science, Ministry of Public Health, Mueang Nonthaburi, Thailand; 4 Yanglum Health Promotion Hospital, Ubonratchathani, Thailand; 5 Department of Microbiology, Faculty of Science, Chulalongkorn University, Bangkok, Thailand; 6 National Institute of Infectious Diseases, Tokyo, Japan

**Keywords:** Vaccines, Preventive medicine

## Abstract

Diabetes mellitus (DM) patients are at an increased risk of complications following influenza-virus infection, seasonal vaccination (SV) is recommended. However, SV with trivalent influenza vaccine (TIV) can induce antibody and type-I interferon (IFN) responses, and the effect of anti-DM treatment on these responses is incompletely understood. We evaluated the antibody response and *IFN-α* expression in individuals with and without type 2 DM (T2DM) following SV, and examined the effects on anti-DM treatment. TIV elicited sero-protection in all groups, but antibody persistency was <8 months, except for the antibody response to B-antigens in non-DM. T2DM impaired the IgG avidity index, and T2DM showed a significantly decreased response against H1N1 and H3N2, in addition to delaying and reducing haemagglutination-inhibition persistency against influenza B-antigens in DM groups treated with metformin (Met-DM) or glibenclamide (GB-DM). Following TIV, the Met-DM and GB-DM groups exhibited reduced *IFN-α* expression upon stimulation with whole- and split-virion influenza vaccines. Suppression of *IFN-α* expression in the Met-DM group was associated with a reduction in the mechanistic target of rapamycin complex-1 pathway and impaired IgG avidity index. Thus, single-dose TIV each year might not be suitable for T2DM. Our data could aid the development of an efficacious influenza vaccine for T2DM.

## Introduction

The prevalence of type-2 diabetes mellitus (T2DM) is increasing worldwide, particularly in developing countries. In 2017, it was estimated that 451 million people worldwide were living with DM, and this number is expected to increase to 693 million by 2045^1^. Due to multiple impairments of the immune system, patients with DM are more susceptible to infections such as influenza virus infection^2,3^.

Annual influenza vaccination is recommended by the World Health Organization (WHO) and the Advisory Committee on Immunization Practices in the USA to prevent influenza infection^4^. The efficacy of vaccination should be evaluated in patients with T2DM, who are classified as a high-risk group for influenza infection^2,5,6^. Interestingly, it has been reported that anti-DM medications further impair immune responses^7–10^. Metformin—the first-line anti-hyperglycaemic drug for T2DM in Thailand—has been reported to impair the immune response by upregulating the expression of 5′ adenosine monophosphate-activated protein kinases (AMPKs) and inhibiting the mechanistic target of rapamycin (mTOR)-mediated pathway^11,12^. Glibenclamide is another anti-hyperglycaemic agent that has been reported to impair immune responses by decreasing the production of interleukin (IL)-1β and IL-8 and decreasing glutathione levels in polymorphonuclear cells^13^. Furthermore, Kewcharoenwong and colleagues showed that glibenclamide reduced primary human monocyte functions against *Mycobacterium tuberculosis*, such as reducing bactericidal activity and IL-1β expression^9^. Nevertheless, the effect of glibenclamide on the antibody response against the influenza virus is not clear.

Type-I interferons (IFNs) play an important role in the host defence against viral infections by enhancing the antiviral function of adaptive immune cells and antibodies^14,15^. Furthermore, several studies have shown that mTOR, a serine/threonine kinase, supports switching of antibody classes, affinity maturation, and promotes formation of germinal centres in lymphoid tissue^16–18^, in addition to playing an essential role in the induction of type-I IFNs in plasmacytoid dendritic cells (pDCs) through interferon regulatory factor (IRF)7 and toll-like receptor (TLR)7 signalling^19–23^. TLR7 and IFN-α have been shown to have important roles in the induction of antibody-class switching and generation of high-affinity antibody against influenza and other viruses^24–28^.

We aimed to address the impairment of immune response following seasonal vaccination with the trivalent influenza vaccine (TIV) in patients with T2DM and observe the effect of common anti-DM drugs—metformin and glibenclamide—on type-I IFN responses.

## Results

### Seasonal vaccination with TIV elicited sero-protection for all groups, but all treated DM groups showed significantly decreased responses against H1N1 and H3N2, in addition to delaying and reducing **haemagglutination inhibition (**HAI) persistency against influenza B antigen in metformin-DM and glibenclamide-DM

The demographic data revealed no significant difference (p > 0.05) between the two groups with respect to age, sex, or body mass index (BMI). Most people in Thailand who are diagnosed with T2DM are prescribed metformin^29^. Consistent with this strategy, approximately 75% of T2DM patients in the present study were also prescribed metformin (Table 1). Individuals with T2DM in our study were prescribed metformin (12/40; 30%), glibenclamide (10/40; 25%), or a combination of both drugs (18/40; 45%). In addition, the longest duration of anti-diabetic drug medication occurred for individuals with DM who were prescribed glibenclamide. However, satisfactory glucose control, defined by glycated haemoglobin (HbA_1c_) <6.5%, was measured for only 22.5% of individuals with DM. According to vaccination history, 90% of DM patients received an influenza vaccination in previous years (Table 1); this showed that T2DM individuals in Thailand followed the WHO recommendation. Considering all available demographic data for the study subjects, it is likely that there were no age- or BMI-related effects on the antibody response against influenza vaccination^30,31^.

**Table 1:** Demographic characteristics of individuals participating in this study.

Demographic		DM (n = 40)	non-DM (n = 30)	P
Age (years)	Median (range)	55 (38–69)	53 (37–70)	0.1539
Sex (%)	Female	68	77	ND
Body mass index (kg/m^2^)	Median (range)	25 (18–36)	25.1 (18–36.5)	0.8308
Fasting blood sugar (mg%)	Median (range)	128.5 (89–435)	90.5 (55–137)	<0.0001
HbA_1c_ (%)	<6.5	22.5	ND	ND
	6.5–8.4	37.5		
	>8.5	40		
Type of anti-diabetic medication	n (%)			ND
Metformin only		12 (30)		
Glibenclamide only		10 (25)		
Metformin + glibenclamide		18 (45)		
Duration of anti-diabetic medication (months)	median (range)			
Metformin only		29 (9–48)		0.0008^a^,
Glibenclamide only		91.5 (36–228)		ns^b^,
Metformin + glibenclamide		33.5 (20–137)		0.0337^c^
History of influenza vaccination	(%)			
previously vaccinated		90	10	ND
not previously vaccinated		10	90	

DM: diabetes mellitus; ND: not determined; HbA_1c_: glycated haemoglobin.

Statistical analyses were undertaken using the unpaired *t*-test, ns: non-significant, ^a^metformin *vs*. glibenclamide, ^b^metformin *vs*. metformin + glibenclamide, ^c^glibenclamide *vs*. metformin + glibenclamide.

**Figure 1:**
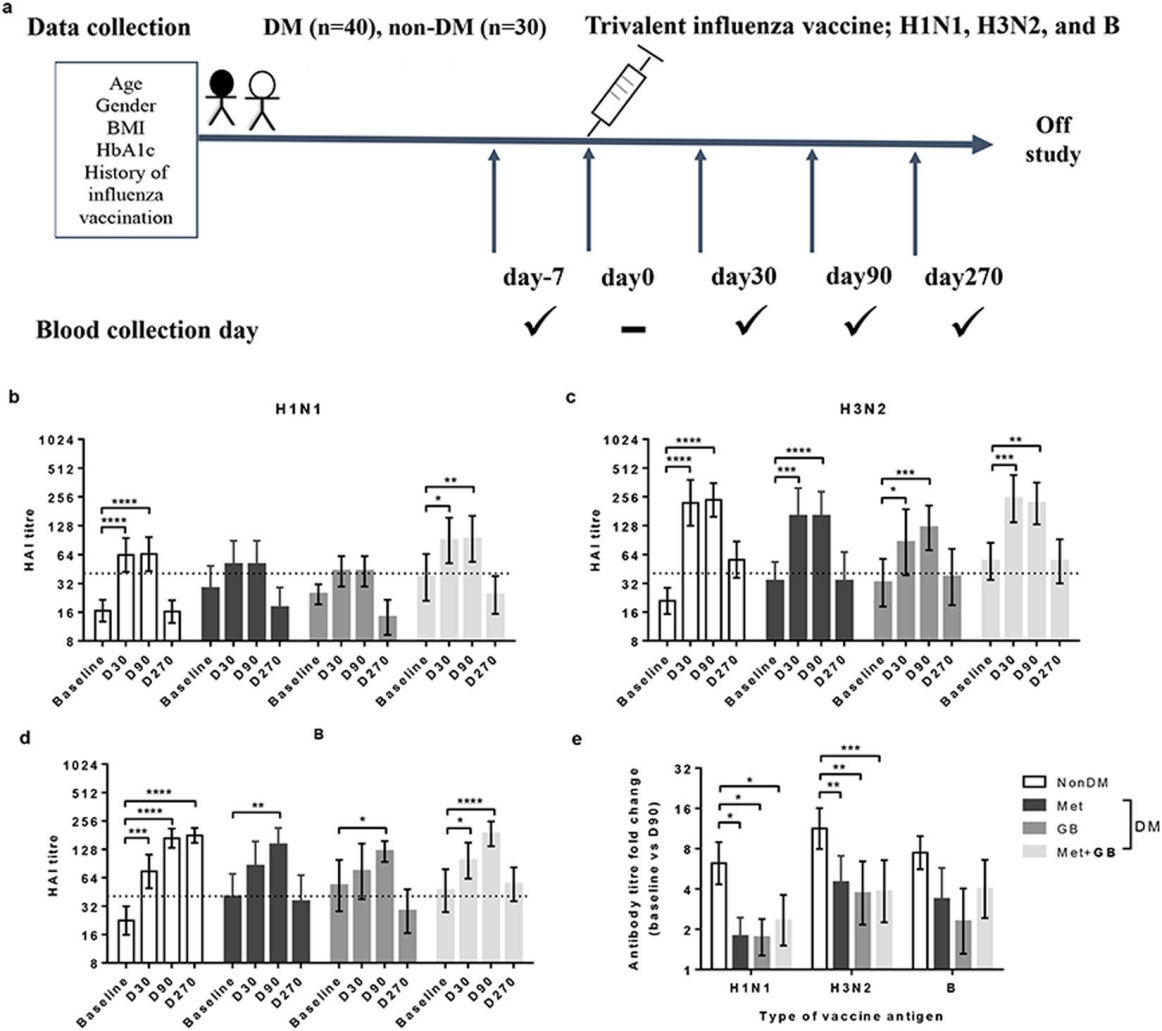
Seasonal vaccination with TIV elicited sero-protection for all groups, but all treated DM groups showed significantly decreased responses against H1N1 and H3N2, in addition to delaying and reducing haemagglutination inhibition (HAI) persistency against influenza B antigen in metformin-DM and glibenclamide-DM. **(a)** Study design indicating the sample groups and time of blood collection before and after seasonal vaccination with TIV (schematic). Blood samples were collected from Thai individuals with and without T2DM at baseline (day -7) and post-vaccination with TIV (day 0). Antibody responses were evaluated by the HAI assay at baseline, as well as 30, 90, and 270 days post-vaccination against influenza **(b)** H1N1, **(c)** H3N2, or **(d)** B-antigen and compared with non-DM individuals and T2DM patients prescribed metformin (Met; n = 12) or glibenclamide (GB; n = 10), or a combination of the two drugs (Met + GB; n = 18). The dotted line represents the protective threshold HAI titre of 40. **(e)** HAI antibody titre fold-change (baseline *vs*. D90) against influenza H1N1, H3N2, or B-antigens compared among all four groups. Comparison of percentage (%)Statistical analyses were undertaken using one-way (**b–d**) or two-way (**e)** ANOVA. Horizontal lines represent the geometric mean with 95% CI. *p < 0.05; **p < 0.01; ***p < 0.001; ****p < 0.0001.

Samples of heparinized blood from patients with T2DM (n = 40) and those without DM (non-DM; n = 30) were collected 7 days before vaccination and on the day of vaccination, as well as 30, 90, and 270 days after vaccination (Fig. 1a). There was no significant increase in the HAI titre against H1N1 at 30 and 90 days as compared with baseline titres in individuals with DM who were prescribed metformin or glibenclamide, except for those without DM and DM patients who had been prescribed a combination of both drugs (Fig. 1b). Conversely, a significant (p < 0.05) increase in the HAI titre against H3N2 at 30 and 90 days compared with that at baseline was observed in all groups (Fig. 1c). However, we found a delayed response in the HAI titre against the influenza B antigen, wherein the maximum response was recorded at 90 days instead of 30 days post-vaccination in DM patients prescribed metformin or glibenclamide (Fig. 1d). Subsequently, the antibody levels decreased 270 days after vaccination (Fig. 1b–d). In other words, TIV could elicit antibody persistency >8 months after vaccination, but the persistency of the antibody response to B antigen in non-DM individuals seemed to be maintained at the same level for >8 months (Fig. 1d).

In addition, fold-change values of the HAI titre (baseline vs. D90) in each DM group were significantly (p < 0.05) lower than those in non-DM group for all types of influenza antigens, except B antigen (Fig. 1e). This finding implied that T2DM significantly decreased responses against H1N1 and H3N2. A recent study indicated that a history of influenza vaccination is a confounding factor for high baseline antibody titres and affects antibody fold-change^32^. To exclude such factors, we chose only those individuals who received an influenza vaccination in the recent year to analyse and compare the HAI titre at baseline, and we compared the HAI fold-change at day 90 with that at baseline (Supplementary Fig. S1a–b). We found that the baseline levels were no different between non-DM and DM groups. However, individuals with DM (who received all types of anti-DM treatment) showed a significantly (p < 0.05) lower fold-change in the HAI antibody titre (baseline *vs*. D90) against H3N2 influenza antigens than non-DM individuals (Supplementary Fig. S1b).

According to guidelines set by the US Food and Drug Administration, sero- response and the sero-protection following influenza vaccination can be defined by a minimum of a fourfold increase and a serum HAI titre >1:40, respectively^33^. After vaccination using TIV, individuals with DM (who received all types of anti-DM medication) showed lower percentages of a sero-response (Supplementary Fig. S1c) against all influenza antigens than those without DM, and in non-DM individuals who received seasonal vaccination with TIV during the previous year. In contrast with sero-response, sero-protection was found in most individuals (Supplementary Fig. S1d), and there was no difference between the proportion of sero-protection in individuals with and without DM. Interestingly, both groups of non-DM individuals (who had or did not have seasonal vaccination with TIV in the previous year) showed a similar sero-response and sero-protection against all types of influenza vaccine antigens (Supplementary Fig. S1e). In addition, to clarify that HAI titre fold-change was not affected by the fact that 90% of individuals in T2DM groups, but only 10% of controls (non-DM), had a history of influenza vaccination, we separated individuals with or without T2DM into previously vaccinated (individuals who had a history of influenza vaccination) or not previously vaccinated (individuals who had no history of influenza vaccination) groups. Then, we compared HAI titre fold-change (baseline versus day 90-post vaccination) between the two groups. There were no significant differences between the previously- and not previously-vaccinated groups in both individuals with or without T2DM (Supplementary Fig. S2). This finding implied that a history of influenza vaccination was a confounding factor for high baseline antibody titres, but may not affect antibody fold-change or sero-protection in individuals with or without T2DM.

### DM impaired the immunoglobulin (Ig) G avidity index

**Figure 2:**
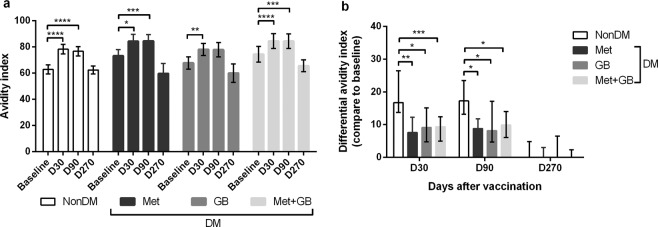
T2DM impaired the fold-change and differential avidity index of IgG. **(a)** The avidity index of influenza-specific IgG antibody at several time points or **(b)** differential avidity index of an influenza-specific IgG antibody response compared with baseline and D30 or D90 or D270 post-vaccination in all individuals. Statistical analyses were undertaken using two-way ANOVA. Horizontal lines represent (**a**) the mean with 95% CI or (**b**) the median with interquartile range. *p < 0.05; **p < 0.01; ***p < 0.001; ****p < 0.0001.

A significant (p < 0.05) increase in the IgG avidity index was observed against all types of influenza vaccine antigens at 30 and 90 days as compared with baseline values in all groups (Fig. 2a). However, there were no differences in the IgG avidity index at several time points among the four groups (non-DM, Met-DM, GB-DM and Met+GB-DM), between individuals who received seasonal vaccination with TIV in the previous year (Supplementary Fig. S3a), or between individuals who had never received seasonal vaccination with TIV (Supplementary Fig. S3b). Nevertheless, individuals with DM who had been prescribed metformin or glibenclamide showed a delayed and low response of the IgG avidity index, respectively (Supplementary Fig. S3c). The prevalence of a differential IgG avidity index at 30, 90, and 270 days compared with that at baseline in all individuals with DM was significantly (p < 0.05) lower than that in those without DM (Fig. 2b). In addition, we chose only individuals who received seasonal TIV vaccination in the previous year to compare differences in the IgG avidity index among the four groups. We found that individuals with DM showed a significantly lower differential IgG avidity index than non-DM individuals at all time points (Supplementary Fig. S3d).

Although there was a difference in vaccination history between the DM and non-DM groups, the fold-change in antibody levels or differential IgG avidity index in those with or without a vaccination history was similar (Supplementary Fig. S1–S3, Supplementary Table S1). This finding implied that previous vaccination against influenza did not affect the antibody response to the current vaccination. In addition, the fold-change in the HAI titre against all types of influenza-vaccine antigens and fold-change in the IgG avidity index in individuals with DM were negatively correlated with the duration of anti-diabetic drug medication (Supplementary Fig. S4). Our multivariate analysis revealed that a duration of anti-diabetic drug medication >40 months (adjusted odds ratio [OR]: 11.127; 95% confidence interval (CI), 1.461–84.732; p = 0.020) was significantly associated with an impaired sero-response to seasonal vaccination with TIV in individuals with T2DM (Supplementary Table S1). Multivariate analysis also showed no effect of HbA_1c_ levels on the sero-response (Supplementary Table S1). This observation was consistent with the data from two previous studies^30,31^.

We observed an impairment in antibody levels, avidity maturation, and a delayed antibody response after vaccination in T2DM individuals. In addition, the HAI titre against the three influenza antigens and fold-change in the IgG avidity index in individuals with T2DM were negatively correlated with the duration of anti-DM medication (Figs. 1 and 2, Supplementary Figure S1–S4, and Supplementary Table 1). Hence, factors such as anti-DM medication in T2DM may affect the immune response against TIV.

### *IFN-α* expression decreased in metformin- and glibenclamide-treated DM groups upon stimulation with whole- and split-virion influenza vaccines

Type-I IFN plays an important role in the antibody response and protection against viral infection^14,34,35^. Type-I IFN is involved in isotype switching of antibodies to orchestrate (together with TLR signalling) production of the appropriate anti-influenza B-cell responses^24,36^. Several recent studies have reported that treatment with anti-DM medications (e.g., metformin and glibenclamide) affects *IFN-α* expression.

As seen in Figs. 1 and 2, anti-DM medication affect the antibody response against TIV. To explore the effect of anti-DM medication on vaccination efficacy, *IFN-α* expression was studied in whole blood cultures (*in vitro*) and compared among groups: non-DM; newly diagnosed T2DM but yet to start treatment with anti-DM drugs (new-DM); Met-DM; and GB-DM. All participants underwent seasonal vaccination with TIV 90 days before being enrolled in our study.

We used three stimuli to represent different TLR agonists for *IFN-α* mRNA expression *in vitro*. The first stimulus was polyinosinic:polycytidylic acid (poly I:C), which is a synthetic analogue of double-stranded RNA (dsRNA), and is usually employed to simulate viral infection *via* the TLR3/RIG-I (retinoic acid-inducible gene I) agonist^37,38^. The second stimulus was the whole-virion vaccine against the influenza (X31, H3N2) virus. This represents the response to natural influenza infection. It retains its particulate structure along with internal single-stranded RNA (ssRNA) and ligands for endosomal TLR7/8^39,40^. The final stimulus was a split-virion influenza vaccine (seasonal TIV). This represents the response to seasonal influenza vaccination. It is a disrupted viral protein lacking a particulate structure and internal ssRNA^36^ or ligands for endosomal TLR7/8^39,40^.

**Figure 3:**
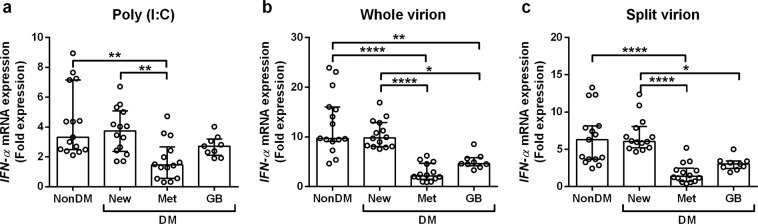
Metformin- and glibenclamide-treated DM reduced *IFN-α* expression upon stimulation with whole- and split-virion influenza vaccines. *IFN-α* expression was determined in whole-blood samples after 3-h stimulation *in vitro* with (**a**) poly I:C, (**b**) influenza virus (influenza whole-virion, X31), or (**c**) influenza vaccine (influenza split-virion) before RNA extraction for measurement of *IFNA1* mRNA expression by real-time PCR. Expression is shown as fold expression with respect to the medium control and normalized to that of *GAPDH*. Each point represents an individual (non-DM; n = 15), (new-DM; n = 14), (Met-DM; n = 14), or (GB-DM; n = 9). Statistical analyses were performed using one-way ANOVA. Horizontal lines represent the median and interquartile range. *p < 0.05; **p < 0.01; ****p < 0.0001.

Expression of *IFN-α* mRNA is shown as fold expression in relation to medium control and normalized to that of the glyceraldehyde 3-phosphate dehydrogenase (*GAPDH*) gene. Significantly lower expression of *IFN-α* upon stimulation with poly I:C only was found in Met-DM individuals as compared with non-DM and new-DM individuals. *IFN-α* expression after stimulation with poly I:C only in GB-DM samples exhibited no significant differences (p > 0.05) with that in non-DM and new-DM individuals (Fig. 3a). These data suggested that T2DM individuals undergoing metformin treatment had an impaired response through the TLR3/RIG-I agonist, but this was not the case in GB-DM or new-DM groups. With regard to the other aspects of stimulation with whole- or split-virion influenza vaccines, similar patterns of *IFN-α* expression were observed: the Met-DM and GB-DM groups showed significantly lower (p < 0.05) expression than that of new-DM and non-DM groups (Fig. 3b–c). These results suggested that impaired expression of *IFN-α* through TLR3/RIG-I activation was exclusively an effect of metformin treatment, but not of glibenclamide treatment. Although robust activation by whole- or split-virion influenza vaccines resulted in impaired expression of *IFN-α* in the Met-DM and GB-DM groups, the effect was more potent in the case of Met-DM.

Previous studies have shown no significant correlation of HbA_1c_ levels with sero-protection for influenza vaccines^31^, even though HbA_1c_ tends to reduce the sero-response or sero-protection against influenza vaccines^30^. We wished to ascertain if low expression of *IFN-α* in T2DM patient prescribed anti-DM medications (metformin or glibenclamide) occurred because of the anti-DM medications rather than glycaemic-control status. Therefore, HbA_1c_ levels and other demographic characteristics of all 4 DM groups were measured upon collection of blood samples, and we found no significant differences (p > 0.05) among the new-DM and other groups of individuals with T2DM (Supplementary Table S2). This finding strongly suggested that the reduction in *IFN-α* expression against the influenza vaccine was due to metformin or glibenclamide rather than the HbA_1c_ level.

### Metformin (but not glibenclamide) reduced *IFN-α* expression in human monocytes

**Figure 4:**
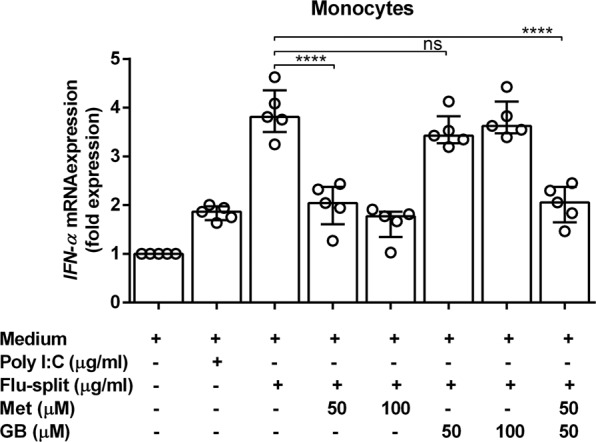
Metformin (but not glibenclamide) reduced *IFN-α* expression in human monocytes after seasonal vaccination. Monocytes from individuals without DM (non-DM; n = 5) were treated with metformin (Met) or glibenclamide (GB) or a combination of both for 30 min. Drug-treated monocytes were incubated with poly I:C or TIV (influenza split-virion) for 3 h before RNA extraction and determination of *IFN-α* expression by real-time PCR. Expression is shown as fold expression. Each point represents an individual. Statistical analyses were undertaken using one-way ANOVA. Horizontal lines represent the median with interquartile range. ****p < 0.0001; ns, not significant.

Metformin- and glibenclamide-treated DM patients exhibited reduced *IFN-α* expression upon stimulation with whole- and split-virion influenza vaccines, but only metformin had a TLR3/RIG-I-induced effect upon *IFN-α* expression (Fig. 3). To demonstrate the effect of anti-DM medication *in vitro*, first, human monocytes were studied from individuals without DM. Human monocytes were pre-treated with or without metformin or glibenclamide at different doses before being stimulated with or without a split virion vaccine. Then, *IFN-α* mRNA expression was detected by real-time polymerase chain reaction (PCR). Metformin (but not glibenclamide) reduced *IFN-α* expression in human monocytes in a dose-dependent manner (Fig. 4).

### Metformin inhibits activation of mTOR complex 1 (mTORC1) on primary human peripheral blood mononuclear cells (PBMCs), resulting in reduced expression of *IFN-α*

**Figure 5:**
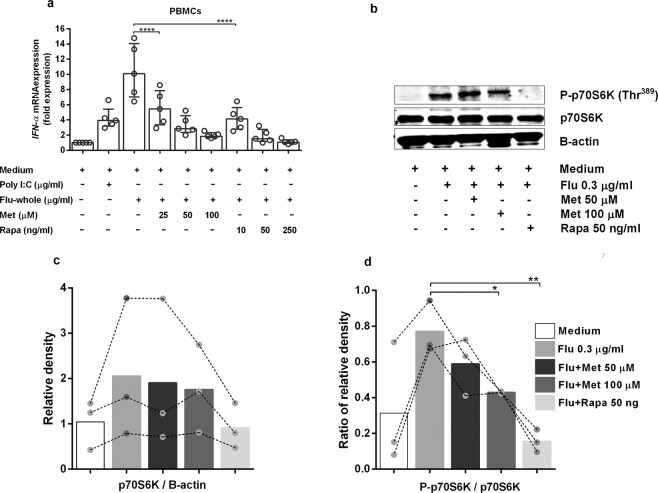
Metformin reduced *IFN-α* expression in primary human PBMCs *via* the mTORC1 pathway. **(a)** Peripheral blood mononuclear cells (PBMCs) from non-DM individuals (n = 5) were treated with metformin (Met) or rapamycin (Rapa) for 30 min. Drug-treated PBMCs were incubated with poly I:C or influenza whole-virion for 3 h before RNA extraction. *IFN-α* mRNA expression was determined by real-time PCR. Expression is shown as fold expression. Each point represents an individual. Statistical analyses were undertaken using one-way ANOVA. Horizontal lines represent the median and interquartile range. **(b)** The same PBMC samples were treated with Met or Rapa for 30 min before incubation with influenza whole-virion for 3 h, followed by determination of total p70S6k and P-p70S6K (Thr^389^) by western blotting. The data are representative of three individuals. **(c)** Relative density of p70S6K. **(d)** Ratio of relative density of P-p70S6K/p70S6K. Each colour bar represents each condition. Each dot represents an individual. The bar graph represents mean values. Statistical analyses were carried out using two-way ANOVA, *p < 0.05, **p < 0.01, ****p < 0.0001.

Researchers have reported that mTORC1 signalling plays an essential role in the induction of type-I IFNs^19–23^. Rapamycin is a potent inhibitor of mTOR signalling^41,42^. To address whether metformin reduces *IFN-α* expression as a response to the influenza virus through inhibition of the mTORC1 pathway, we pre-treated human PBMCs with or without various doses of metformin or rapamycin before stimulation with a whole-virion vaccine or poly I:C. Then, we measured *IFN-α* expression by real-time PCR, and found that metformin and rapamycin reduced *IFN-α* expression in human PBMCs in a dose-dependent manner (Fig. 5a). This finding suggested that metformin and rapamycin follow a similar pattern in reduction of *IFN*-α expression.

To investigate the effect of metformin on mTORC1 signalling in greater detail, healthy human PBMCs were pre-treated with or without metformin or rapamycin before stimulation with the medium control or whole-virion vaccine. Phosphorylated p70S6K (Thr^389^), p70S6K (Thr^389^), and β-actin protein from cell-culture lysates were detected by western blotting. Metformin and rapamycin reduced phosphorylated levels of p70S6K (Thr^389^) (Fig. 5b and Supplementary Fig. S5). The relative density and ratio of the relative density of western blotting were also determined (Fig. 5c–d). Metformin did not change p70S6K expression, but reduced the phosphorylation level of this protein. Taken together, these results suggested that metformin reduced *IFN-α* expression upon influenza stimulation through inhibition of mTORC1 signalling by reducing the phosphorylation of p70S6K (Thr^389^). Furthermore, *IFN-α* expression from exposure to a split-virion influenza vaccine was positively correlated with the IgG level and fold-change in the IgG avidity index (Supplementary Fig. S6).

## Discussion

Several studies have shown that a four-fold increase in the HAI titre is a useful predictor for the efficacy of an influenza vaccine^43–45^ and that T2DM does not impair the antibody response^30,31,46^. However, our data showed that approximately 80% of T2DM cases did not have impaired sero-protection, but instead showed an impaired sero-response, fold-change in HAI titre and IgG avidity index, and a significantly decreased response to H1N1 and H3N2, as well as delayed and reduced responses of antibody persistence to the influenza-B antigen in those prescribed metformin or glibenclamide. We also found that the duration of anti-DM medication reduced the sero-response and avidity for the influenza-specific IgG antibody. These findings showed that seasonal vaccination with TIV was most likely to have been efficacious in fewer than half of the Thai individuals with T2DM we assessed. Our findings imply that T2DM status or treatment with anti-DM drugs may affect the response against seasonal vaccination using TIV.

We also found higher baseline antibody levels and reduced antibody persistence to the influenza-B antigen (Phuket strain, which is a new strain) in T2DM individuals prescribed metformin or glibenclamide. Unfortunately, we could not verify this effect in the present study. Although 90% of individuals with T2DM and only 10% of those without T2DM had previously undergone vaccination for influenza, the sero-response did not differ greatly between the two groups. Also, we found a similar pattern of HAI titre or avidity of IgG antibody in all individuals or only in influenza-vaccinated individuals. Trieu and colleagues suggested that a history of influenza vaccination was a confounding factor for high baseline antibody titres and affected fold-changes in antibody levels^32^. This conflicting finding could be attributed to the low numbers of individuals with T2DM who did not undergo seasonal vaccination with TIV in the previous year, and the very low numbers of people without T2DM who previously underwent seasonal vaccination with TIV. Individuals with DM belong to one of the seven high-risk groups who received the recommendation to obtain seasonal vaccination with TIV, whereas individuals without DM need not necessarily undergo vaccination^5^. Therefore, most non-DM individuals in our study did not previously undergo influenza vaccination.

We also found that seasonal vaccination with TIV could elicit an antibody response at >60 days, with a gradual decline by 270 days post-vaccination. This finding showed that seasonal vaccination with TIV could elicit antibody persistence approximately 8 months after vaccination except in non-DM individuals who continued to show an antibody response to influenza-B antigen >270 days post-vaccination. However, given that an outbreak of seasonal influenza occurs in annual cycles (usually in rainy and winter months) in temperate climates^47^, the Ministry of Public Health (MOPH) in Thailand recommends vaccinating individuals once a year. Therefore, two doses of the influenza vaccine may be required, especially for high-risk groups, which includes people with T2DM prescribed metformin or glibenclamide.

Our findings also showed that the duration of use of anti-DM medication reduced the avidity index of influenza-specific IgG antibody. This finding supports the notion of a negative effect of glucose-control medication on the antibody response following influenza vaccination in individuals with T2DM. However, the findings of the present study are not in accordance with those that found no effect of treatment duration^30,31,46^.

IFN-α expression is important in limiting the initial influenza infection because IFN-α drives the adaptive immune response^14,15,35,39^. Measurement of IFN-α expression is also useful for predicting the efficacy of an influenza vaccine^48,49^. Therefore, we also aimed to determine the effect of different hypoglycaemic drug treatments on *IFN-α* expression *in vitro* in DM, non-DM, and new-DM groups. However, IFN-α can be activated through various TLR agonists, such as TLR3/RIG-I or TLR7/8, as recognized with dsRNA or ssRNA, respectively^39,40^. Therefore, we used different TLR agonists: poly I:C (dsRNA) for a representative IFN-α response through TLR3/RIG-I, and TLR7/8 by two formulae of influenza (ssRNA) vaccine (antigen); whole-virion influenza vaccine (representing the natural response to infection); and split-virion influenza vaccine (representing the response to seasonal vaccination using TIV). This is the first report to show an impaired IFN-α response *via* TLR3/RIG-I and TLR7/8 in T2DM patients prescribed metformin, whereas only *via* TLR7/8 in those prescribed glibenclamide. We could not determine the IFN-α response between non-DM and new-DM groups for both TLR agonists. This finding likely implies that metformin and glibenclamide can suppress IFN-α expression through a TLR agonist. In addition, studies have shown that IFN-α production by antigen-presenting dendritic cells in lymphoid tissue and the pancreas is lower in patients with T2DM than in non-DM individuals^5,50^.

We found that metformin and glibenclamide reduced *IFN-α* expression in the whole blood of individuals who received seasonal vaccination using TIV, but we did not observe a corresponding reduction of *IFN-α* expression in human monocytes when pre-treated with glibenclamide. This finding is in accordance with studies that have reported a reduction in IL-1β expression from the primary human monocytes of DM patients treated with glibenclamide against *Mycobacterium bovis* Bacillus Calmette Guérin^9^ and a reduction of type-I IFN production by human monocyte-derived macrophages against *M. tuberculosis* in the presence of IL-1^10^. Taken together, these findings suggest that glibenclamide does not reduce IFN-α production against *M. tuberculosis* by monocytes. It is possible that, because of these reasons, we could not find a reduction in *IFN-α* expression from human monocytes when they had been pre-treated with glibenclamide. Although glibenclamide has been shown to increase the production of type-I IFN from human monocytes against *Mycobacterium* species^9,10^, little is known about the effect of glibenclamide on IFN-α production following influenza vaccination in people with T2DM, and further studies will be required to determine this effect.

Researchers have shown that mTORC1 signalling is important in TLR-mediated IFN-α responses in pancreatic dendritic cells^19,22,23,51,52^. In addition, the main function of metformin is to reduce hepatic glucose production by activating AMPK, which results in inhibition of mTOR signalling and a subsequent decrease in gluconeogenesis^11^. Moreover, glibenclamide, another drug used to treat T2DM, also has an effect on human immune responses^13^ and inhibits AMPK activation^53^, resulting in enhanced mTOR signalling. Nonetheless, the direct effect on influenza-virus response is not clear. We found that metformin reduced *IFN-α* expression of human PBMCs against the influenza virus *via* the mTORC1 pathway. Furthermore, our results showed a positive correlation between *IFN-α* expression and fold-changes in influenza-specific IgG antibody and the avidity index of influenza-specific IgG antibody. However, our findings are also in accordance with several studies that found the IFN-α production by dendritic cells in human T2DM to be lower than that in non-DM individuals^5,50^. These observations suggest that IFN-α plays a role in enhancing and regulating antibody affinity, and they correlate with studies that highlighted the important role of mTORC1 in the antibody response^17,18,54^. In addition, studies have shown that IFN-α enhances B-cell class switching^24^, and promotes a neutralizing antibody response against virus infection^55^. Reduced *IFN-α* expression *via* decreased mTORC1 signalling in individuals with T2DM who were prescribed metformin may lead to reduced selection of germinal centres and affinity maturation.

Our results also showed greater immunogenicity for the innate immune response to a whole-virion influenza (X31) vaccine than a split-virion influenza vaccine. This finding is consistent with studies that found a higher type-1 IFN response against a whole-virion vaccine than a split-virion vaccine^25,36,56^. Therefore, our results may be useful in improving the immunogenicity of split-virion influenza vaccines using pancreatic dendritic cell-activating adjuvants that could improve the intrinsic TLR7 signalling in B cells^25,36,56^.

## Conclusions

Seasonal vaccination with TIV boosted sero-protection in most T2DM and non-DM individuals. However, T2DM caused an impaired sero-response, affinity fold-change, which is a significantly decreased response against H1N1 and H3N2, in addition to delaying and reducing HAI persistency against influenza-B antigens, especially in patients prescribed metformin or glibenclamide. Our findings may imply that healthy individuals who are vaccinated with the influenza vaccine undergo induction of p70S6K phosphorylation (Thr^389^), downregulation of the mTORC1 signalling pathway (which promotes *IFN-α* expression), and induction of antibody-class switching or affinity maturation, thereby leading to effective control of viral infection. However, in T2DM patients prescribed metformin, we found that metformin inhibited p70S6K phosphorylation (Thr^389^). This action led to reduced *IFN-α* expression and, consequently, affected influenza-specific IgG-antibody and avidity index responses, which may have affected the efficacy of viral control. This finding supports the current recommendations that T2DM patients should receive an annual influenza vaccination. However, annual, single-dose, seasonal vaccination with TIV may not be suitable for people with T2DM, and especially those prescribed metformin. We believe that these findings may be useful for developing an efficient influenza vaccine for high-risk groups (especially T2DM patients) and for achieving increased immunity in these patients.

## Methods

### Ethical approval of the study protocol

The study protocol was carried out in accordance with the approved guidelines by the Khon Kaen University Ethics Committee on Human Research (HE592038; Khon Kaen, Thailand), and all subjects provided written informed consent after the study had been explained to them.

### Study participants and seasonal vaccination with TIV

All participants were defined here as aged over 18 years and matched sex and age. None of the participants had any signs of acute infectious disease in the three months and did not receive seasonal vaccination with TIV in the six months prior to vaccination. Moreover, Non-DM group consisted of individuals with normal fasting blood glucose levels (<110 mg%). The healthy individuals were recruited from the hospital stuff, who live in the same endemic area as diabetic individuals. The criteria for exclusion for both Non-DM and DM groups were having a history of immune deficiency, suffering from alcoholism, having an acute illness or signs of illness at the time of vaccination.

Individuals who were diagnosed with T2DM (n = 40) and individuals without DM (non-DM) (n = 30) who attended the Yanglum Health Promotion Hospital (Ubonratchathani, Thailand) were enrolled in the study. The antibody response against TIV was evaluated by measuring the antibody titre using an HAI test. The IgG antibody, or avidity index of IgG antibody, was determined by an enzyme-linked immunosorbent assay (ELISA).

All participants were administered a single dose of TIV (Fluvirin^®^; Novartis, Basel, Switzerland) (season 2015–2016). TIV is a non-adjuvant split-virus composed of H1N1 (California strain), H3N2 (Switzerland strain), and influenza-B virus (Phuket strain). Seasonal vaccination using TIV is supported by the MOPH in Thailand.

The study groups comprised people without DM (non-DM), those that were newly diagnosed (new-DM), individuals with T2DM who were prescribed metformin (Met-DM), and those with T2DM who were prescribed glibenclamide (GB-DM). Duration of anti-diabetic drug use was denoted from when the T2DM patient began anti-diabetic drug until the date of drawing the blood. All groups were included in the evaluation of *IFNA1* expression. All participants were vaccinated with TIV 90 days before being enrolled in the study.

### Vaccine-specific antibody titre

HAI titres were used to determine vaccine-specific antibody titres based on a standard WHO protocol, as previously described^57^. Briefly, plasma samples were treated with receptor-destroying enzyme (RDE) (Denka Seiken, Tokyo, Japan) by adding one-part plasma to three-parts RDE and incubating at 37 °C overnight. The next day, RDE was inactivated by incubating the samples at 56 °C for 1 h. Then, the samples were serially diluted with phosphate-buffered saline (PBS) in 96-well V-bottom plates (Nalge Nunc International Corporation, Rochester, NY, USA), and 4 HA-units each of H1N1 (California strain), H3N2 (Switzerland strain), or influenza-B virus (Phuket strain) was added to each well. After 30 min at room temperature, 50 µl of 0.5% turkey red blood cells (Rockland Immunochemicals, Philadelphia, PA, USA) suspended in PBS with 0.5% bovine serum albumin (BSA) was added to each well, and the plates were manually agitated. After an additional 30 min at room temperature, the plasma titres were read. Negative and positive control plasma samples for each virus were used for reference. “Sero-protection” was defined as an HAI titre >1:40, and a “sero-response” was defined as a minimum four-fold increase in antibody titre, 30 days post-vaccination^33^.

### Measurement of influenza-specific IgG antibody and differential avidity index

Influenza-specific IgG antibody and IgG avidity index were determined for each patient before and after vaccination, as previously described^58^. Briefly, ELISA plates were coated with TIV (H1N1, H3N2, B-antigens) at 100 ng/well. After blockade with 1% BSA in PBS, all sera were added to four-fold serial dilutions in duplicate and incubated at room temperature for 2 h. Plates were washed five times with 0.1% Tween-20 in PBS (PBST). Bound IgG was detected with horseradish peroxidase-conjugated antibodies specific to human IgG-Fc (Southern Biotech, Birmingham, AL, USA) at room temperature for 1 h. The plates were washed five times with PBS, and then, 100 μl of *O-*phenylenediamine dihydrochloride substrate was added. The reaction was terminated with 1 M H_2_SO_4_, and the optical density (OD) was measured at 450 nm. The avidity index was determined by modifying the immunoassay with the addition of 7 M urea (treated) or PBS (untreated) and incubating for 30 min before the detection step. The avidity index of the antibody was represented as the differential avidity index (% urea resistance at day 30 or day 90 or day 270, minus with % urea resistance at baseline). The % urea resistance was calculated based on the dose–response curves and compared between with and without-urea treatments.

### Measurement of *IFNA1* expression from whole blood

Briefly, 10^7^ lymphocytes/well from the whole blood of each patient were plated in 96-well culture plates and cultured with or without TIV by adding 0.3 μg/ml of influenza whole-virion or split-virion or 50 μg/ml of poly I:C for 3 h before sample collection into Tempus™ RNA tubes (Applied Biosystems, Foster City, CA, USA). RNA was extracted using a Tempus Spin RNA Isolation kit (PN4378926) (Applied Biosystems) and cDNA conversion by an ImProm-II^™^ (Promega, Madison, WI, USA) reverse-transcription system according to the manufacturer’s instructions.

*IFNA1* expression was determined by real-time PCR carried out using a Light Cycler^®^ 96 system (Roche, Basel, Switzerland) with commercially available fluorescein amidite (FAM)-labelled probes (ACCAGTTCCAGAAGGCTCCAG [oligo number 8021382436-000090] and FastStart Essential DNA Probe Master (Roche Molecular Diagnostics, Pleasanton, CA, USA; catalogue number 06402682001).

The following forward and reverse primers, respectively, were used: CCCAGGAGGAGTTTGATG (oligo number 8021859939-000010) and CCAAGCAGCAGATGAATC (oligo number 8021859939-000020) (Sigma Life Sciences, Singapore). *GAPDH* was used for normalization. ΔCt values (Ct of the target gene minus the Ct of *GAPDH*) for each double sample were averaged, and ΔΔCt was calculated. mRNA amplification was determined by the formula 2^−ΔCt^, as previously described^59^. The relative quantification of mRNA levels was plotted as the relative expression of stimulated samples minus that of non-stimulated samples, or fold expression relative to the medium control (divided by the relative expression of non-stimulated samples). *GAPDH* was used with commercially available FAM-labelled probes (TCCACGACGTACTCAGCGCC) (oligo number 8021382436-000080), FastStart Essential DNA Probe Master Mix (Roche Molecular Diagnostics; catalogue number 06402682001), and primers (forward: CCATCTTCCAGGAGCGAGATCC [oligo number 8021382436-000030] and reverse: ATGGTGGTGAAGACGCCAGTG [formerly oligo number 8021382436-000040]) (Sigma Life Sciences).

### Isolation of PBMCs and monocytes

PBMCs and monocytes were isolated from the heparinized venous blood of individuals without DM by dextran sedimentation using Ficoll-Hypaque density gradient centrifugation and Lymphoprep^™^ (STEMCELL Technologies, Vancouver, BC, Canada: catalogue number 07851), as previously described^60^. The cell viability was >98%, as determined by the trypan blue exclusion assay. We isolated monocytes from PBMCs using the plastic adherence method, as previously described^60^. The resulting cell preparation was confirmed to comprise >85% monocytes by a five-part differential white blood cell analyser. Monocyte morphology was determined by microscopy.

### Measurement of *IFN-α* expression from primary human PBMCs or monocytes

Briefly, 3 × 10^6^ PBMCs/well from non-DM individuals were plated in 48-well culture plates and pre-treated with metformin (Met; 0, 50, 100 μM) (D150959-5G; Sigma-Aldrich, Saint Louis, MO, USA) or rapamycin (Rapa: 0, 10, 50, 250 ng/mL) (AdipoGen Life Sciences, Liestal, Switzerland; catalogue number 53123-88-9) for 30 min before being stimulated with or without influenza whole-virion (0.3 μg/ml) or poly I:C (50 μg/ml) (VacciGrade^™^ 10 mg; catalogue number “vac-pic”; InvivoGen, San Diego, CA, USA) for 3 h. Next, RNA was extracted to determine *IFNA1* expression by real-time PCR as described above.

For monocyte experiments, 7 × 10^5^ monocytes/well from non-DM individuals were plated in 48-well culture plates and pre-treated with metformin (0, 50, 100 μM) or glibenclamide (0, 50, 100 μM) (G0639-5G; Sigma-Aldrich) for 30 min before being stimulated with or without influenza split-virion (Flu-split; 0.3 μg/ml) or poly I:C (50 μg/ml) for 3 h before RNA extraction to determine *IFNA1* expression by real-time PCR as described above. The relative quantification of mRNA levels was plotted as the fold increase over non-stimulated samples (fold-expression).

### Measurement of mTORC1 signalling by western blotting

The same human PBMC samples that were pre-treated with metformin (0, 50, 100 μM) or rapamycin (0, 50 ng/mL) for 30 min before being incubated with or without influenza whole-virion (0.3 μg/ml) for 3 h were used to determine p70S6k and P-p70S6K (Thr^389^) by western blotting.

Protein extracts from cultured cells were washed with cold PBS and lysed with lysis buffer according to the manufacturer’s instructions. Cell lysates were centrifuged at 3000 × *g* for 5 min at room temperature. Supernatants were collected for western blotting, which was carried out using anti-rabbit antibodies (catalogue number 8885) against phosphorylated p70S6k (Thr^389^) (108D2) rabbit monoclonal antibody (catalogue number 9234) and p70S6k (49D7) rabbit monoclonal antibody (catalogue number 2708) (all from Cell Signaling Technology, Danvers, MA, USA).

Equal amounts (30 μg) of total protein in each cell lysate were separated by sodium dodecyl sulphate-polyacrylamide gel electrophoresis (SDS-PAGE) using 10% gels and transferred to polyvinylidene difluoride (PVDF) membranes. The latter were blocked with 3% skimmed milk in PBST and incubated overnight at 4 °C with the corresponding primary antibodies in 3% dried skimmed milk powder in PBST, followed by incubation with horseradish peroxidase-conjugated secondary antibodies for 1 h at room temperature. Signals were detected using the chemiluminescence method. The density of western blot bands was quantified with ImageJ software (National Institutes of Health, Bethesda, MD, USA). Then, the relative density of the test proteins was calculated by dividing the band density with β-actin (loading control). The ratio of the relative density was calculated by dividing the relative density of P-p70S6K by the relative density of p70S6K.

### Statistical analysis

Statistical analyses were carried out using one-way and two-way analysis of variance (ANOVA) and *post hoc* testing with the Bonferroni correction. We calculated the power of each test with 95% CIs, and >80% was acceptable for all experiments. Correlation analysis was performed using Pearson’s correlation coefficient (r). Linear regression was determined by the goodness of fit (R^2^). Non-parametric tests of significance were performed if a normal distribution could not be assessed or if populations did not have a normal distribution. PRISM 6 software (GraphPad, San Diego, CA, USA) was used for all statistical analyses; p < 0.05 was considered significant.

## Supplementary information

Supplementary Figures and Tables.
