# Supplementary material for: Metformin-induced suppression of IFN-α via mTORC1 signalling following seasonal vaccination is associated with impaired antibody responses in type 2 diabetes
Source: Sci Rep. 2020 Feb 24;10:3229. doi: 10.1038/s41598-020-60213-0 (PMC7039947; doi:10.1038/s41598-020-60213-0)
Supplement: Supplementary file 1 — Supplementary Figures and Tables. [file 41598_2020_60213_MOESM1_ESM.pdf]

Supplementary information

Metformin-induced suppression of *IFN-α* via mTORC1 signalling following seasonal vaccination is associated with impaired antibody responses in type 2 diabetes

Wipawee Saenwongsa[1,2], Arnone Nithichanon[1], Malinee Chittaganpitch[3], Kampaew Buayai[3], Chidchamai Kewcharoenwong[1], Boonyarat Thumrongwilainet[4], Patcharavadee Butta[5], Tanapat Palaga[5], Yoshimasa Takahashi[6], Manabu Ato[6] and Ganjana Lertmemongkolchai[1,*]

[1]Centre for Research and Development of Medical Diagnostic Laboratories, Faculty of Associated Medical Sciences, Khon Kaen University, Thailand.

[2]Disease Prevention and Control Region 10th, Ubonratchathani, Ministry of Public Health, Thailand.

[3]National Influenza Centre, Department of Medical Science, Ministry of Public Health, Thailand.

[4]Yanglum Health Promotion Hospital, Ubonratchathani, Thailand.

[5]Department of Microbiology, Faculty of Science, Chulalongkorn University, Bangkok, Thailand.

[6]National Institute of Infectious Diseases, Tokyo, Japan.

***Corresponding author**: Ganjana Lertmemongkolchai, PhD. Cellular and Molecular Immunology Unit, Centre for Research and Development of Medical Diagnostic Laboratories, Faculty of Associated Medical Sciences, Khon Kaen University, Khon Kaen 40002, Thailand. Tel: +66 4320 3825; fax: +66 4320 3826; email: ganja_le@kku.ac.th

Diseases, Tokyo, Japan.

Supplementary Figures S1-S6

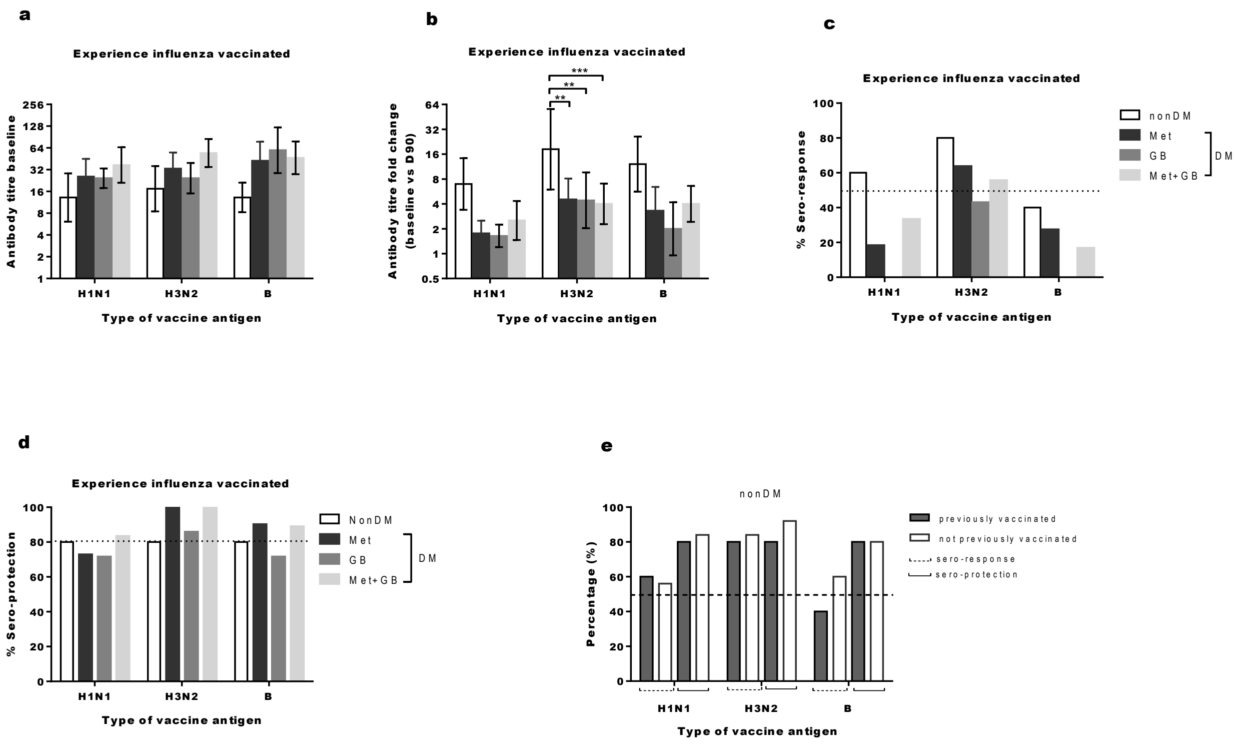

Supplementary Figure S1: History of influenza vaccination was a confounding factor for a higher baseline (but not significantly so) in DM and seemed not to enhance the HAI response in DM and non-DM.

(**a**) The HAI titre at baseline against three types of influenza vaccine antigens in influenza-vaccinated individuals; non-DM (n = 5), Met-DM (n = 11), GB-DM (n = 7), Met+GB-DM (n = 18). (**b**) HAI titre fold-change (baseline *vs.* D90) in influenza-vaccinated individuals among the four groups. Horizontal lines represent the geometric mean with 95%CI. Statistical analyses were undertaken using two-way ANOVA, $**p<0.01$, $***p<0.001$. (**c**) Sero-protection or (**d**) sero-response in people who received seasonal vaccination using TIV among the four groups. The dotted line represents 80% or 50% of sero-protection or sero-response, respectively. (**e**) Percentage (%) of non-DM who reached a sero-response or sero-protection against three type of influenza vaccine antigens compared with individuals who had a history of influenza vaccination (previously vaccinated; n = 5) or who had no history of influenza vaccination (not previously vaccinated; n = 25).

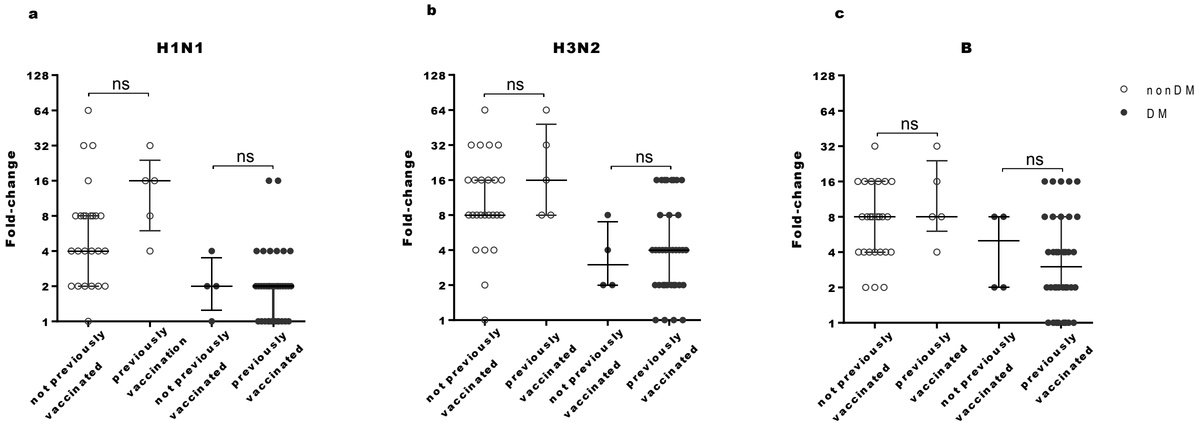

Supplementary Figure S2: History of influenza vaccination seemed not to enhance HAI titre fold-change in DM and non-DM.

HAI titre fold-change (baseline *vs.* D90) in individuals who had a history of influenza vaccination (previously vaccinated, non-DM; n = 5, DM; n = 36) or had no history of influenza vaccination (not previously vaccinated, non-DM; n = 25, DM; n = 4) against **(a)** H1N1, **(b)** H3N2, or **(c)** B antigens. Horizontal lines represent the median with the interquartile range. Each point represents an individual. Statistical analyses were undertaken using Mann–Whitney test. ns; not significant ($p \geq 0.05$), ****$p<0.0001$.

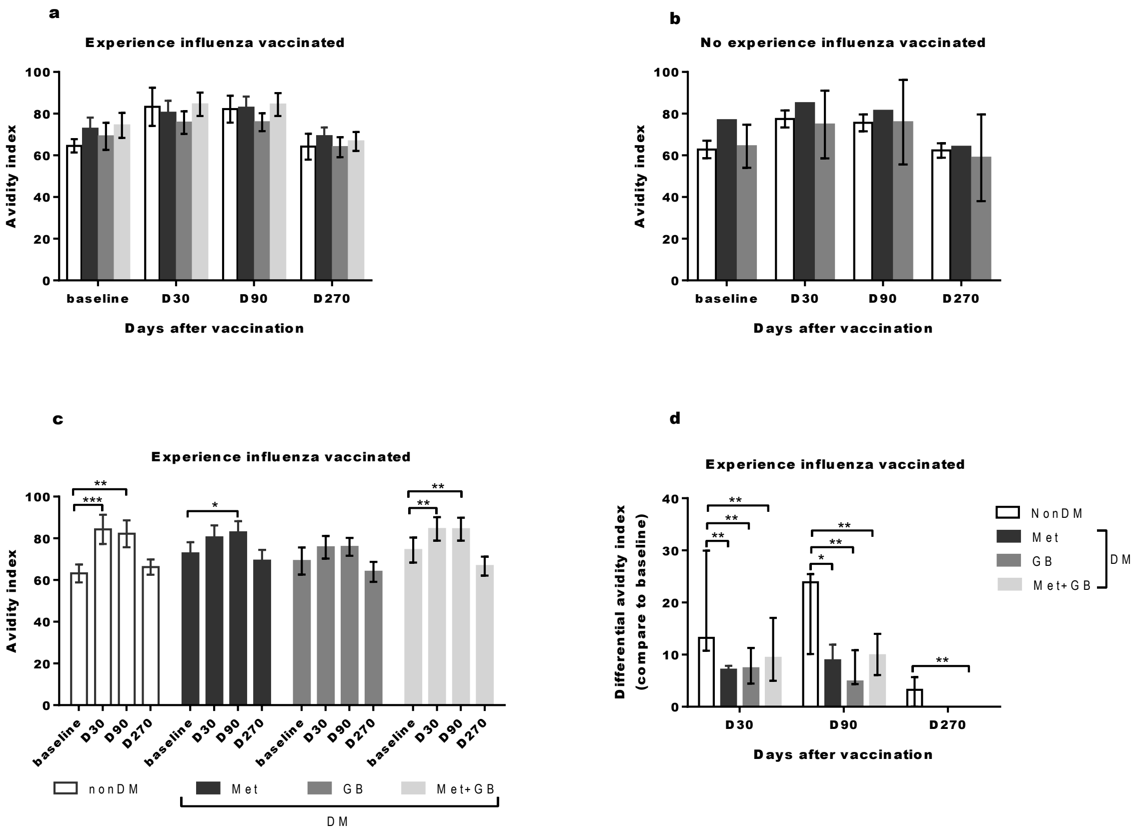

Supplementary Figure S3: The influenza-specific IgG avidity index at baseline and several time points post-vaccination was not significantly different among non-DM and DM, but Met-DM delayed the response of the IgG avidity index, whereas GB-DM decreased the response of the IgG avidity index.

Non-DM and DM individuals (**a**) with (non-DM; n = 5, Met-DM ; n = 11, GB-DM; n = 7, Met+GB-DM; n = 18) or (**b**) without (non-DM; n = 25, Met-DM; n = 1, GB-DM; n = 3) experience of seasonal influenza vaccination were determined, and the influenza-specific IgG avidity index at baseline and several time points post-vaccination were compared using ELISAs. (**c**) The IgG avidity index at baseline and several time points post-influenza vaccination in individuals who had experience of influenza vaccination. (**d**) Differential avidity index of an influenza-specific IgG antibody response compared with baseline as well as D30 or D90 or D270 post-vaccination in those who received seasonal vaccination with TIV. Horizontal lines represent the mean with 95%CI. Statistical analyses were undertaken using two-way ANOVA, *p<0.05, **p<0.01, ***p<0.001.

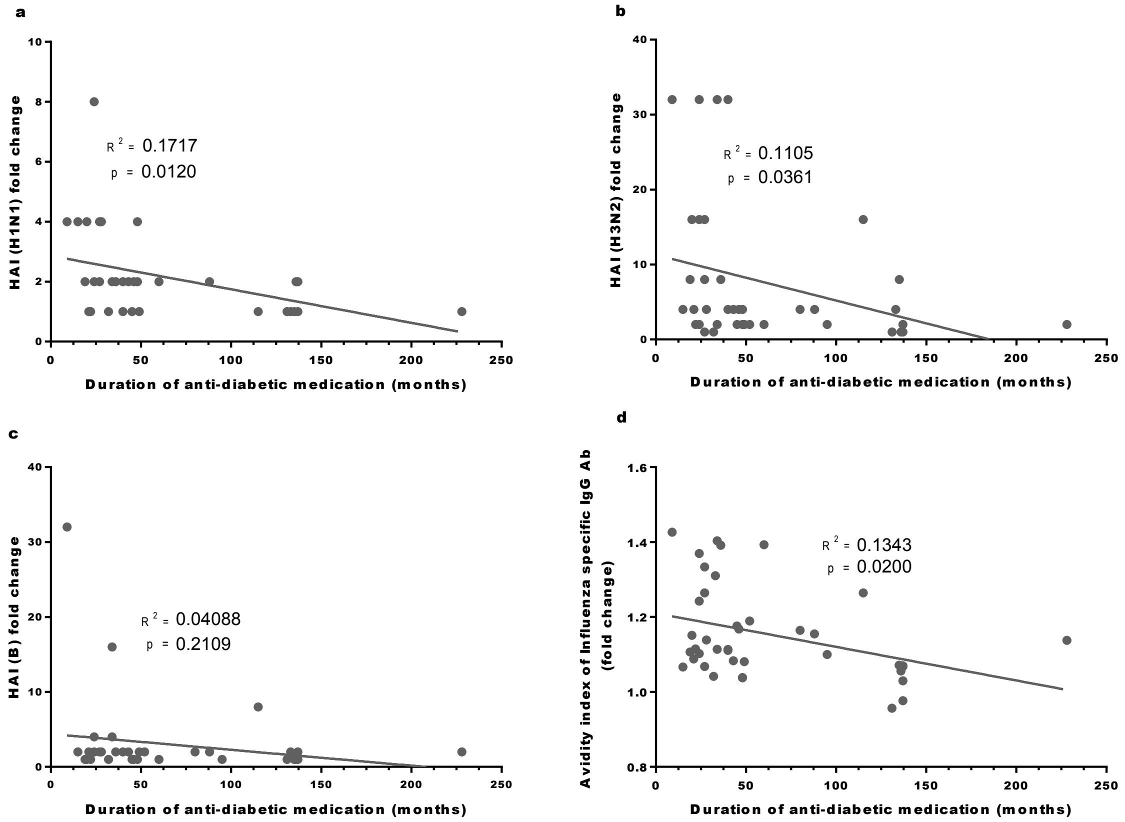

Supplementary Figure S4: Prolonged treatment reduced HAI antibody fold-changes and the avidity index of influenza-specific IgG antibody against seasonal TIV.

The correlation between HAI antibody fold-changes in response to (**a**) H1N1, (**b**) H3N2), (**c**) B antigens, or (**d**) the avidity index of influenza-specific IgG antibody fold-change and duration of anti-diabetic medication (months). The fold change of the response was calculated by dividing the response at D30 by that at baseline (D-7). Each point represents a DM individual (n = 40). R^2 and p represent the Pearson correlation coefficient and associated p-value, respectively.

a No. 1

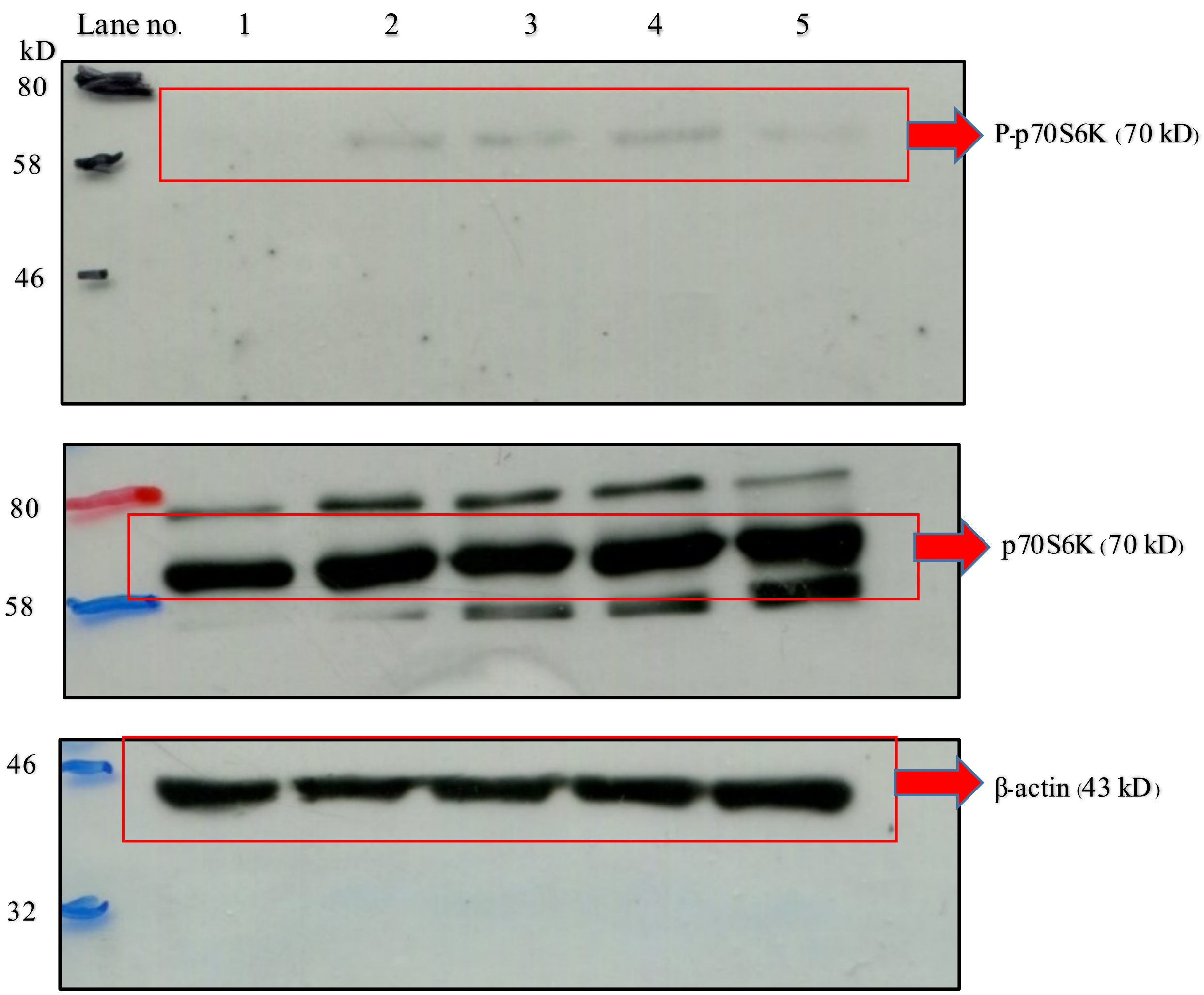

Lane no.
1
2
3
4
5
kD
80
58
46
P-p70S6K (70 kD)
80
58
p70S6K (70 kD)
46
32
β-actin (43 kD)

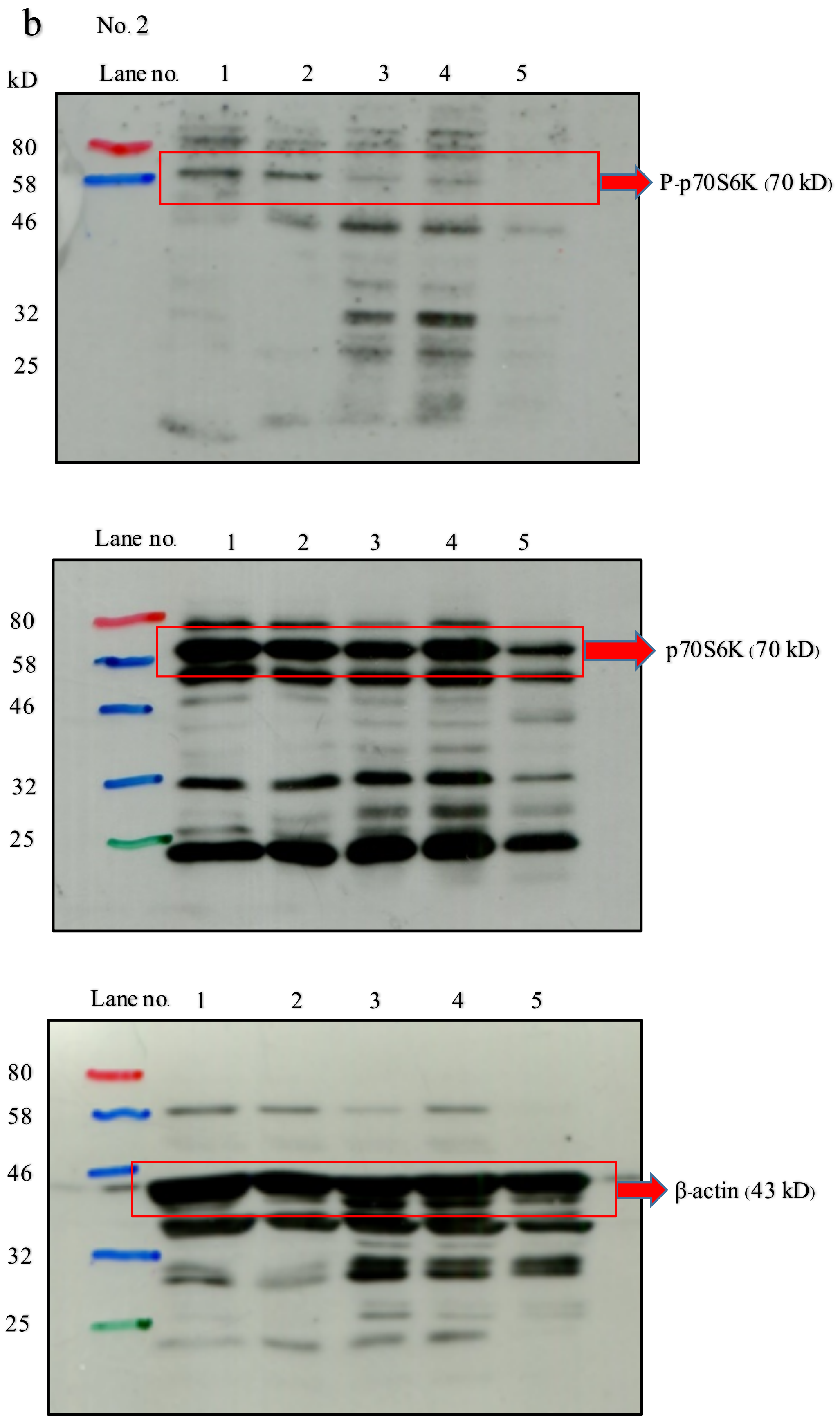

b
No. 2
kD
Lane no. 1 2 3 4 5
80
58
46
32
25
P-p70S6K (70 kD)
Lane no. 1 2 3 4 5
80
58
46
32
25
p70S6K (70 kD)
Lane no. 1 2 3 4 5
80
58
46
32
25
β-actin (43 kD)

C No. 3

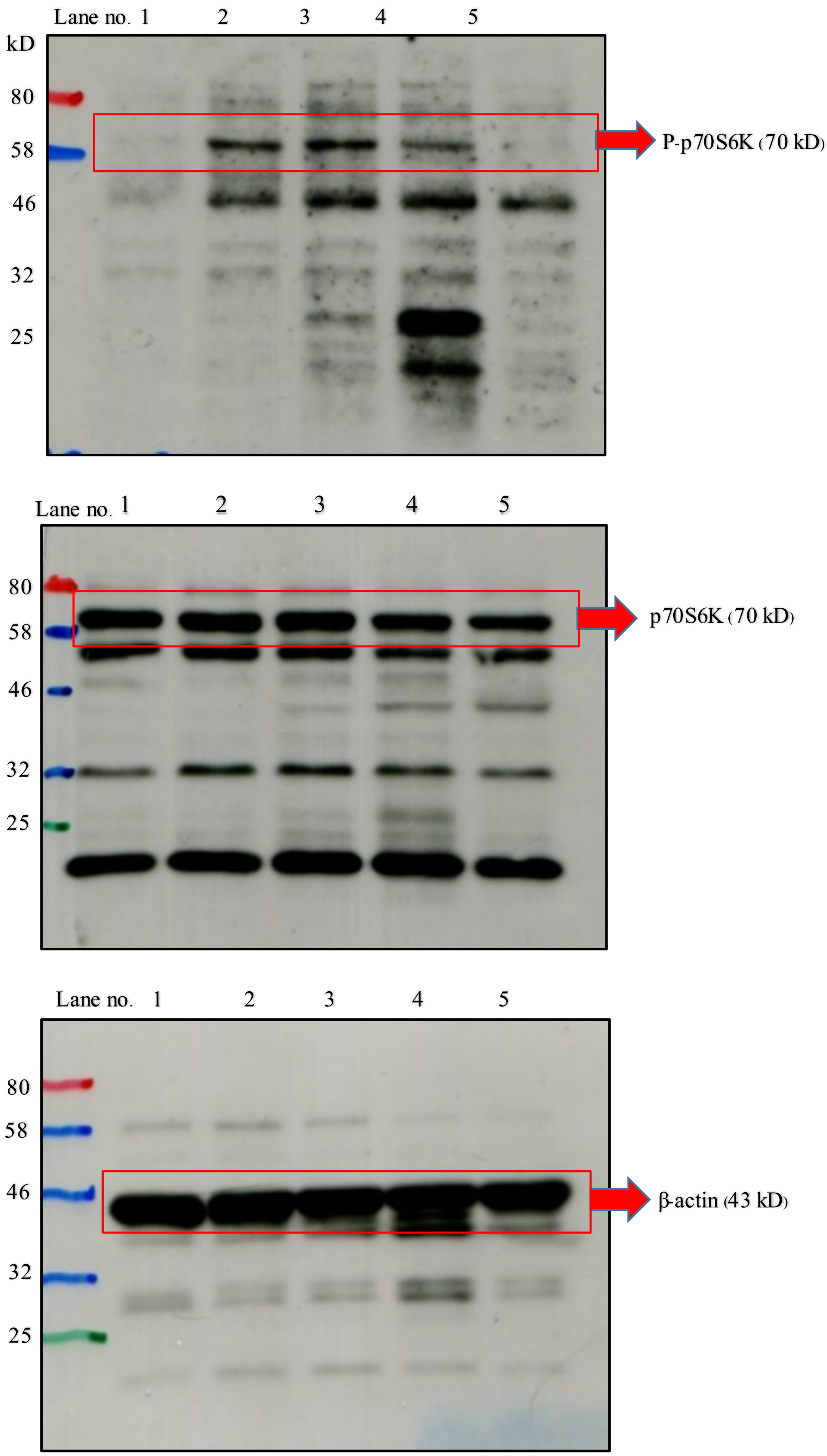
Lane no. 1 2 3 4 5
kD
80
58
46
32
25
P-p70S6K (70 kD)
Lane no. 1 2 3 4 5
80
58
46
32
25
p70S6K (70 kD)
Lane no. 1 2 3 4 5
80
58
46
32
25
β-actin (43 kD)

Supplementary Figure S5: Full-length blots of samples of human PBMCs.

Samples of PBMCs from three non-DM individuals (a–c) were separated into five conditions: medium control (lane number 1), stimulated with influenza whole-virion (lane number 2), treated with Met (50 μM) (lane number 3), Met (100 μM) (lane number 4) or rapamycin (50 ng/mL) (lane number 5) before stimulation with influenza whole-virion for 3 h. Protein was extracted from cultured cells, and P-p70S6K and p70S6K determined by western blotting. β-actin was used as a loading control.

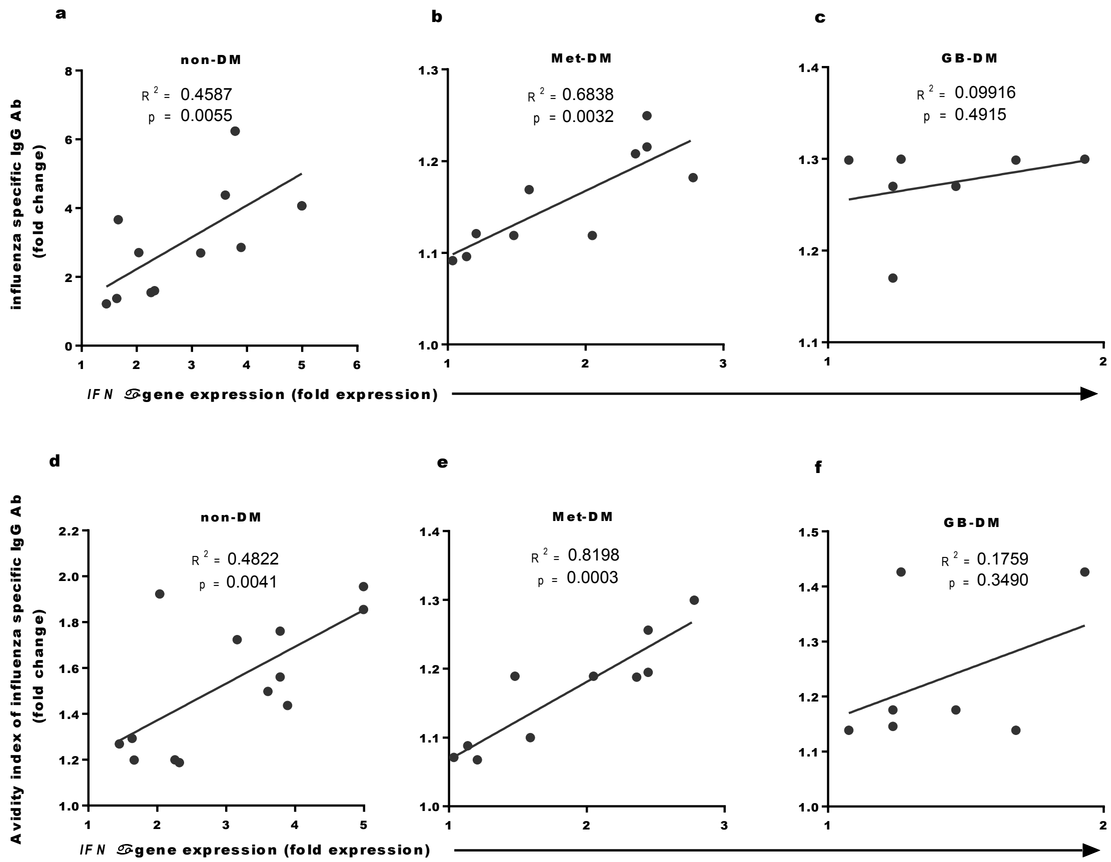

Supplementary Figure S6: Influenza-specific IgG antibody and the avidity index of the influenza-specific IgG antibody response were positively correlated with *IFN-α* expression in non-DM individuals and DM individuals treated with metformin.

Using the same subjects as those shown in Figure 2, non-DM (n = 15) or DM individuals who had been treated with metformin (Met-DM, n = 10) or glibenclamide (GB-DM, n = 7) had blood samples collected 90 days post-vaccination. *IFN-α* expression against an influenza vaccine (influenza-split virion, 0.3 µg/ml) *in vitro* was determined by real-time PCR. A correlation was shown between *IFN-α* expression (fold expression) and (**a**) the influenza-specific IgG antibody response (fold change at D-7/D30) in non-DM or (**b**) in Met-DM individuals or (**c**) in GB-DM individuals and (**d**) in the avidity index of influenza-specific IgG antibody (fold change at D-7/D30) in non-DM or (**e**) in Met-DM individuals or (**f**) in GB-DM individuals. Each point represents an individual. R^2 and p represent the Pearson correlation coefficient and associated p-value, respectively.

Supplementary Table S1

Supplementary Table S1: Multivariate analysis of the host factors that affect the immune response to seasonal trivalent influenza vaccine

Category	N	OR	95%CI	P
HAI (H1N1) fold rise ≥4 (D-7/D30)				
DM	40			
Non-DM	30	4.631	0.261–82.260	0.296
Age ≥60 years	17			
Age <60 years	53	3.201	0.598–17.113	0.174
Female	50			
Male	20	0.517	0.068–3.910	0.523
BMI ≥30 kg/m^2	7			
BMI <30 kg/m^2	63	0.215	0.022–2.049	0.182
HbA_{1c} <6.5%	9			
HbA_{1c} 6.5-8.4%	15	3.057	0.349–26.743	0.313
HbA_{1c} ≥8.5%	16	0.858	0.086–8.523	0.897
Duration of drug use ≥40 months	22			
duration of drug use <40 months	18	11.127	1.461–84.732	0.020
Not previously vaccinated against influenza	30			
Previously vaccinated against influenza	40	1.492	0.209–10.652	0.690

N = number of individuals, OR = odds ratio, CI = confidence interval, Likelihood ratio, logistic regression was used for analyses

Supplementary Table S2

Supplementary Table S2: Demographic characteristics of individuals participating in the study

Demographic		non-DM	new-DM	Met-DM	GB-DM	Met+GB-DM	P
Age (years)	Median (range)	53 (49–58)	52 (39–67)	52 (38–64)	56 (44–69)	55 (42–67)	a–j ns
Sex (%)	Female	77	68	62	70	65	ND
BMI (kg/m^2)	Median (range)	23.4 (19.5–33.5)	26.4 (23.4–33.2)	24.6 (19.9–33.8)	24.9 (19.4–29.1)	25.6 (18.3–30.7)	a–j ns
FBS (mg%) baseline	Median (range)	90.5 (55–137) n = 30	ND	121 (95–285) n = 12	126 (89–376) n = 10	134.5 (98–435) n = 18	a–d**, h–j ns
FBS (mg%) day90	Median (range)	83.5 (68–105) n = 15	279 (180–325) n = 14	137.5 (96–217) n = 14	118.5 (95–335) n = 9	123.5 (84–427) n = 18	a****, b***, c***, d**, e–j ns
HbA_{1c} (%) baseline	Median (range)	ND	ND	7.5 (5.3–11.7), n = 12	8.2 (6.4–11.6), n = 10	7.9 (5.1–11.9), n = 18	h–j ns
HbA_{1c} (%) day 90	Median (range)	5.5 (4.7–5.9) n = 15	8.2 (7.5–10.2) n = 14	7.3 (5.8–10.6) n = 14	7.4 (6.2–9.8) n = 9	7.9 (5.7–11.4) n = 18	a****, b****, c***, d****, e–j ns

DM: diabetes mellitus; ND: not determined: FBS: fasting blood sugar.

Statistical analyses were done using one-way ANOVA. ns: non-significant;
[a] non-DM *vs.* new-DM
[b] non-DM *vs.* Met-DM
[c] non-DM *vs.* GB-DM
[d] non-DM *vs.* Met+GB-DM
[e] new-DM *vs.* Met-DM
[f] new-DM *vs.* GB-DM
[g] new-DM *vs.* Met+GB-DM
[h] Met-DM *vs.* GB-DM
[i] Met-DM *vs.* Met+GB-DM
[j] GB-DM *vs.* Met+GB-DM
*p<0.05, **p<0.01, ***p<0.001, ****p<0.0001
